# A 1.5-Mb continuous endogenous viral region in the arbuscular mycorrhizal fungus *Rhizophagus irregularis*

**DOI:** 10.1093/ve/vead064

**Published:** 2023-10-31

**Authors:** Hongda Zhao, Ruixuan Zhang, Junyi Wu, Lingjie Meng, Yusuke Okazaki, Hiroyuki Hikida, Hiroyuki Ogata

**Affiliations:** Chemical Life Science, Institute for Chemical Research, Kyoto University, Gokasho, Uji 611-0011, Japan; Chemical Life Science, Institute for Chemical Research, Kyoto University, Gokasho, Uji 611-0011, Japan; Chemical Life Science, Institute for Chemical Research, Kyoto University, Gokasho, Uji 611-0011, Japan; Chemical Life Science, Institute for Chemical Research, Kyoto University, Gokasho, Uji 611-0011, Japan; Chemical Life Science, Institute for Chemical Research, Kyoto University, Gokasho, Uji 611-0011, Japan; Chemical Life Science, Institute for Chemical Research, Kyoto University, Gokasho, Uji 611-0011, Japan; Chemical Life Science, Institute for Chemical Research, Kyoto University, Gokasho, Uji 611-0011, Japan

**Keywords:** *Nucleocytoviricota*, *Asfarviridae*, endogenous virus, mycovirus, *Rhizophagus irregularis*

## Abstract

Most fungal viruses are RNA viruses, and no double-stranded DNA virus that infects fungi is known to date. A recent study detected DNA polymerase genes that originated from large dsDNA viruses in the genomes of basal fungi, suggestive of the existence of dsDNA viruses capable of infecting fungi. In this study, we searched for viral infection signatures in chromosome-level genome assemblies of the arbuscular mycorrhizal fungus *Rhizophagus irregularis*. We identified a continuous 1.5-Mb putative viral region on a chromosome in *R. irregularis* strain 4401. Phylogenetic analyses revealed that the viral region is related to viruses in the family *Asfarviridae* of the phylum *Nucleocytoviricota*. This viral region was absent in the genomes of four other *R. irregularis* strains and had fewer signals of fungal transposable elements than the other genomic regions, suggesting a recent and single insertion of a large dsDNA viral genome in the genome of this fungal strain. We also incidentally identified viral-like sequences in the genome assembly of the sea slug *Elysia marginata* that are evolutionally close to the 1.5-Mb putative viral region. In conclusion, our findings provide strong evidence of the recent infection of the fungus by a dsDNA virus.

## Introduction

The fungal virosphere is dominated by RNA viruses, and a few single-stranded (ss) DNA viruses have been identified in phytopathogenic fungi (Yu et al. 2010; Li et al. 2020). Fungal viruses (i.e. mycoviruses) have been classified into twenty-three viral families, among which the twenty-two RNA virus families consist of 204 mycovirus species, while the ssDNA virus family comprises 2 mycovirus species (Kondo, Botella, and Suzuki 2022). No double-stranded (ds) DNA virus has been identified in fungi, but recent studies suggest that they exist. A single-virion sequencing study recovered the genomes of dsDNA viruses belonging to the phylum *Nucleocytoviricota* (nucleocytoviruses) from subsurface oceanic crustal fluids, in which *Ascomycota* fungi are the main eukaryotes (Bhattacharjee et al. 2023). These viral genomes had genes that originated in fungi, suggesting that the viruses infect fungi. Additionally, DNA polymerase genes that likely originated in *Nucleocytoviricota* were also identified in the genomes of basal fungi, including *Rhizophagus irregularis*, an arbuscular mycorrhizal fungus (Gong, Zhang, and Han 2020). *Nucleocytoviricota* is a phylum of viruses with large dsDNA genomes (70 kb to 2.5 Mb) (Aylward et al. 2021). Their hosts include diverse eukaryotes, from protists to animals (Schulz, Abergel, and Woyke 2022).

Endogenous viral elements (EVEs) are a form or trace of viral genomes integrated into the host genome (Feschotte and Gilbert 2012). Some previous studies identified genes from nucleocytoviruses in the genomes of eukaryotes (Maumus et al. 2014; Maumus and Blanc 2016; Yoshikawa et al. 2019). A recent study detected giant EVEs (GEVEs) from nucleocytoviruses in the genomes of green algae (Moniruzzaman et al. 2020; Moniruzzaman, Erazo-Garcia, and Aylward 2022). A GEVE may consist of several hundred kilobases or more than a thousand kilobases although it is often scattered across multiple contigs due to fragmented genome assemblies. The integration of nucleocytoviral genomic sequences into host genomes has significant implications for the evolution of eukaryotes, in which up to 10 per cent of the open reading frames (ORFs) may have originated from GEVEs (Moniruzzaman et al. 2020). Such evolutionary events may be associated with the horizontal gene transfer among eukaryotic organisms (Cheng, Wong, and Melkonian 2021). Furthermore, multiple signals of virophages (dsDNA viral parasites of large DNA viruses) were detected in a variety of eukaryotic genomes (Bellas et al. 2023), reflecting the substantial diversity of the uncharacterized dsDNA virosphere in the eukaryotic domain. Analyses of EVEs may be useful for exploring the virosphere of eukaryotes and currently unidentified virus–host relationships. This is supported by previous reports describing *Nucleocytoviricota* genes in the genomes of plants (moss and fern) (Maumus et al. 2014) and oomycetes (Hingamp et al. 2013; Hannat et al. 2021) even though nucleocytoviruses have not been isolated from these organisms.

One of the difficulties in identifying GEVEs is the fragmentation of GEVEs due to the highly fragmented assemblies of eukaryotic genomes. The recently reported chromosome-level *R. irregularis* genome assemblies (Yildirir et al. 2022) enabled us to search for more complete viral fragments in the genome of this species. In the present study, we screened the genomes of five *R. irregularis* strains for *Nucleocytoviricota* signals and identified a 1.5-Mb GEVE region from a *Nucleocytoviricota*-like virus in a chromosome of strain 4401. This is the largest continuous GEVE region that has been identified to date. This GEVE is homologous to the genomes of *Asfarviridae*, a family belonging to *Nucleocytoviricota*. The results of this study provide evidence of dsDNA viruses in the fungal virosphere.

## Results

### Datasets of *R. irregularis*

We collected five chromosome-level *R. irregularis* genome assemblies corresponding to five different strains (Yildirir et al. 2022) (Table 1). These genomes were assembled using data generated by Illumina sequencing, Nanopore sequencing, and high-throughput chromatin conformation capture (Hi-C) sequencing. Long reads generated by Nanopore sequencing may be longer than a repeat or hypervariable region. In addition, Hi-C sequencing detects spatial proximity and is used for scaffolding. These techniques have contributed to the improvement of the genome assemblies and the achievement of complete chromosome data.

**Table 1.** *R. irregularis* genomeic data.

Strain	Genome size (bp)	Number of scaffolds	Isolated place	Accession
A1	147,088,061	33	Switzerland	GCA_020716765.1
B3	146,830,121	33	Switzerland	GCA_020716685.1
DAOM-197198	147,209,168	33	Canada	GCA_020716725.1
C2	161,924,360	33	Switzerland	GCA_020716745.1
4401	146,854,905	33	Canada	GCA_020716705.1

### Continuous 1.5-Mb GEVE region on a fungal chromosome

**Figure 1.**
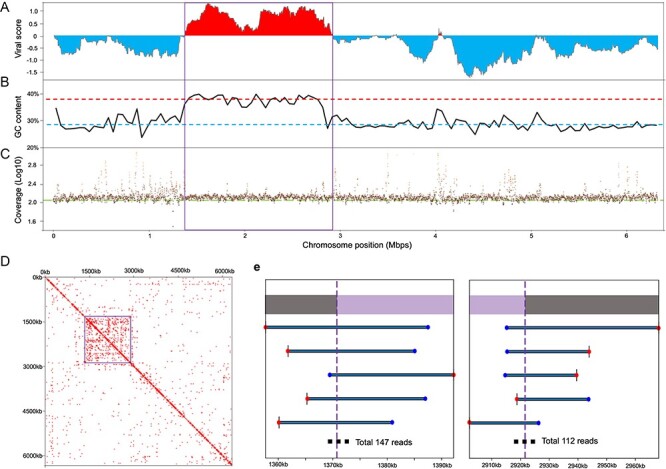
Details regarding the 1.5 Mb GEVE region of the fungal chromosome. (A) ViralRecall score of chromosome 8 in strain 4401. Viral scores were evaluated with a rolling window of 150 ORFs on the chromosome. The score of each ORF was based on the HMM scores of the viral reference database and cellular reference database. High and low scores represent viral and cellular regions, respectively. (B) GC content along the chromosome. The window size is 50,000 bp. The red and blue dashed lines represent the average GC content of the GEVE region (36.58%) and the remaining parts of chromosome 8 (28.00%), respectively. (C) Average read coverages of the chromosome. Each dot represents the average coverage of 1,000 continuous base pairs. The green line represents the average sequencing depth (bases/genome size = 113×). (D) Heat map showing Hi-C signals of chromosome 8. The resolution is 25 kb. Squares with signal values greater than 1 are marked in red and represent high contact probabilities. (A–D) The viral region is indicated by squares. (E) Examples of long reads connecting the GEVE regions identified by ViralRecall with the remaining parts of the chromosome. We present the five longest representative reads. Blue and red points indicate the start and end of the read, respectively. The extremities of the GEVE region are indicated by purple vertical dashed lines (1,370,742 bp in the figure on the left and 2,921,549 bp in the figure on the right). Purple and gray bars at the top indicate the GEVE and other cellular chromosomal regions, respectively.

ViralRecall is a tool designed for detecting viral regions on the basis of the hidden Markov model (HMM) profile of *Nucleocytoviricota* orthologous groups; the function used to identify ten *Nucleocytoviricota* marker genes was integrated into this tool. By using ViralRecall to analyze the five *R. irregularis* genome assemblies, we identified a giant continuous viral signal as a 1,550-kb region (1,370,742–2,921,549 bp) on chromosome 8 of strain 4401 (Fig. 1A, Supplementary Fig. S1).

This 1.5-Mb viral region had distinct sequence and structural features. Of the ten analyzed *Nucleocytoviricota* marker genes, five were included in the 1.5-Mb viral region. These genes encode B-family DNA polymerase (PolB), RNA polymerase large subunit (RNAPL), RNA polymerase small subunit (RNAPS), mRNA capping enzyme (mRNAc), and viral late transcription factor 3 (VLTF3) (Supplementary Table S1, Supplementary Figs S2–S6). The average guanine and cytosine (GC) content of the *R. irregularis* genome was 27.89 per cent, whereas the viral region had a GC content of 36.58 per cent. The GC content throughout the region was also higher than that of the remaining parts of the chromosome (Fig. 1B). Furthermore, the Hi-C sequencing data indicated that the DNA in the region was more condensed than the DNA in other genomic regions (Fig. 1D).

To confirm whether this viral region corresponds to a bona fide insertion in the fungal chromosome, we mapped the raw long reads to the fungal genome. Most chromosomal regions, including the viral region, had similar coverage (Fig. 1C). There were many short regions with a high read coverage, but they mostly corresponded to repetitive elements. There were 259 long reads directly connecting the identified viral region and cellular regions (Fig. 1E). These results confirmed that the viral region represents a GEVE integrated into the fungal chromosome.

In the GEVE region, we identified 1,359 ORFs, which account for 36.22 per cent of the region (Supplementary Table S2). This coding density is much lower than that of other typical nucleocytoviruses (>80 per cent) (Schulz et al. 2020). Of the 1,359 ORFs, 134 were functionally annotated, which revealed that 37, 23, and 15 ORFs were related to ‘replication, recombination, repair’, ‘signal transduction mechanisms’, and ‘transcription’, respectively (Supplementary Fig. S7). Intron-like sequences were found in twenty-two predicted genes (Supplementary Table S2). Eighteen of these genes were annotated in the original gene annotation of the fungal genome. Two of these eighteen genes show homology to viral genes, indicating their viral origins.

Apart from ORFs, we detected forty-eight potential pseudogenes (including RNAPL) distributed throughout the entire viral region. Besides the intergenic region with potential pseudogenes, there were 1,095 intergenic regions that were longer than 100 bp (total length: 907 kb) (Supplementary Fig. S8). To identify the traces of genes in these intergenic regions, we used these 1,095 intergenic sequences as queries for BLASTx searches to the NR database. Of these queries, 124 sequences (11.3 per cent) matched sequences in the protein database (*E*-value < 10^−5^). Most of these matches (121 of the best 124 BLASTx alignments) corresponded to *Rhizophagus* protein sequences in the NR database. Nearly four-fifths of these best hits were annotated as hypothetical proteins or unnamed protein products, making them difficult to conclude the origins (either fungal or viral) of these sequences. We also searched the homology between these 1,095 intergenic sequences and all viral regions we detected in other chromosomes of the five fungal strains. Two hundred and twenty-two intergenic sequences (20.3 per cent) matched ORFs from the GEVEs (*E*-value < 10^–5^). Among them, 74 sequences were included in the 124 sequences with *Rhizophagus* hit, suggesting that some of the 124 sequences are of viral origin. The detection of tandem repeats in the GEVE region using Tandem Repeat Finder indicated that the total length of the tandem repeats in this region was 36 kb (2.32 per cent) (Supplementary Fig. S9).

### The identified GEVE is specific to strain 4401

**Figure 2.**
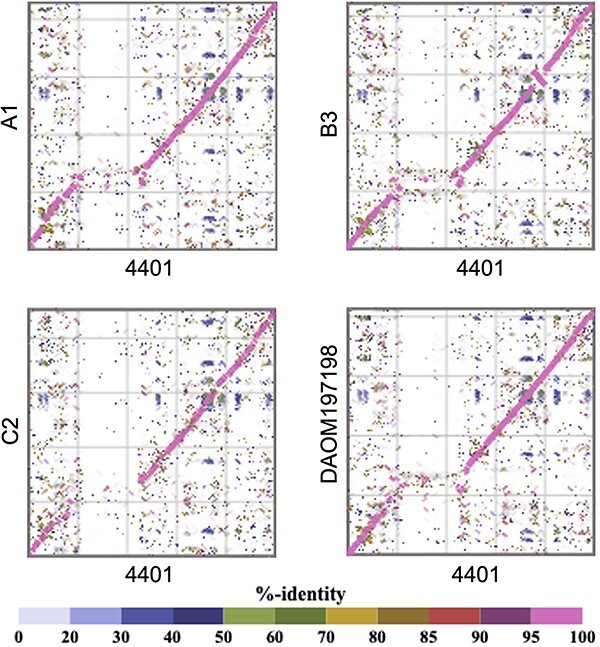
Comparison of chromosome 8 among strains. Each dot represents an amino acid sequence similarity according to tBLASTx.

In all five fungal strains, fifty-nine putative viral regions were detected, including the 1.5-Mb GEVE region (Supplementary Table S3, Supplementary Fig. S10). However, with the exception of the 1.5-Mb GEVE region, all of these regions were shorter than 300 kb and encoded only PolB (fifteen to thirty-three copies per genome) and/or mRNAc (one copy in strains A1 and B3) (Supplementary Table S4). We compared chromosome 8 in the five *R. irregularis* strains in terms of the similarity in the translated sequences (Fig. 2). Most of the strain 4401 chromosome 8 translated sequences were highly similar to the corresponding sequences in the other strains (>90 per cent sequence identity). However, the other strains lacked the region corresponding to the 1.5-Mb GEVE region in strain 4401. Consistent with this finding, chromosome 8 in strain 4401 was revealed to be ∼1 Mb longer than chromosome 8 in the other strains (Table 2). These results indicate that the integration of the GEVE in chromosome 8 is unique to strain 4401.

**Table 2.** Chromosome 8 length in each strain.

Strain	Length (bp)
A1	5,441,258
B3	5,350,159
C2	5,721,097
DAOM-197198	5,236,836
4401	6,327,528

**Figure 3.**
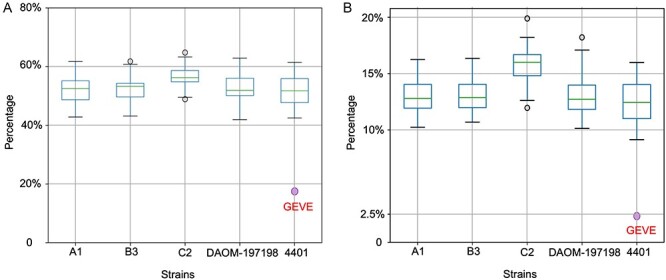
Proportion of the *R. irregularis* chromosomes comprising repeats and TEs. (A) Percentage of the chromosomes of the five strains and the 1.5 Mb GEVE region containing repeating sequences. The purple dot represents the repeat content in the GEVE region (18.28%). (B) Percentage of the chromosomes of the five strains and the 1.5 Mb GEVE region containing TE sequences. The purple dot represents the TE content in the GEVE region (2.31%). These known TEs represent a subset of the identified repeats in (A).

Repeating sequences represent ∼50 per cent (on average) of the genomes of *R. irregularis* strains according to RepeatMasker (Yildirir et al. 2022). In the current study, we determined that 42–65 per cent of the individual chromosomes of the five *R. irregularis* strains consisted of repeats. In contrast, repeats represented only 18.28 per cent of the 1.5-Mb GEVE region (5.37 per cent in the coding region and 12.91 per cent in the non-coding region) (Fig. 3A). The repeats in the non-coding regions accounted for 17.78 per cent of the non-coding regions. An analysis of the repeats related to known transposable elements (TEs) indicated the TE content of the GEVE region (2.31 per cent) was lower than that of the whole genome (average of 13.45 per cent) (Fig. 3B).

### The GEVE is homologous to *Asfarviridae* sequences

**Figure 4.**
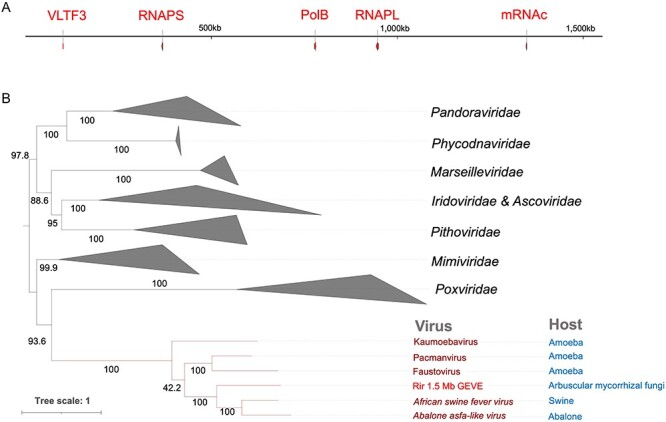
Marker genes in the 1.5 Mb GEVE region. (A) Distribution of *Nucleocytoviricota* marker genes in the 1.5 Mb GEVE region. Marker genes are in red. (B) Concatenated maximum-likelihood phylogenetic tree constructed using three markers (PolB, RNAPL, and RNAPS). Rir, *R. irregularis*. Because of long branch attraction, we manually pruned the clade *Mininucleoviridae*. The root of the tree was arbitrarily chosen and the tree should be considered as an unrooted tree. Ultrafast bootstrap support values are provided along the branches. The best-fit model was Q.pfam+F+I+I+R8.

The five *Nucleocytoviricota* marker genes in the 1.5-Mb GEVE region were detected as single copies and were dispersed in the GEVE region (Fig. 4A). Phylogenetic analyses involving the five marker genes indicated that they are closely related to the homologs from *Asfarviridae* (Supplementary Figs S2–S6). We performed another phylogenetic analysis using a concatenated sequence of the three longest and most universal nucleocytovirus marker sequences (PolB, RNAPL, and RNAPS) (Fig. 4B). In the constructed tree, the GEVE was classified as a sister group of the clade including *African swine fever virus* and *Abalone asfa-like virus* (100 per cent ultrafast bootstrap support).

### Lack of major capsid protein homologs in the fungal genomes

Of the ten analyzed marker genes, the 1.5-Mb GEVE region did not include the genes encoding major capsid protein (MCP), A32-ATPase (A32), D5 primase/helicase (D5), ribonucleotide reductase (RNR), and superfamily II helicase (SFII). These five genes were not detected in the genomes of the five strains. We found homologs of the *Asfarviridae* minor capsid protein and capsid protein H240R encoding genes in the GEVE region (171,954–176,120 bp and 80,214–81,161 bp, respectively). We performed tBLASTn and other searches using HMM models of MCP sequences from multiple viral groups, including typical MCPs of nucleocytoviruses (see ‘Methods’ for details). However, MCP genes were not detected in strain 4401 or the other four strains.

### The fungal GEVE is closely related to virus-like sequences from a sea slug

**Figure 5.**
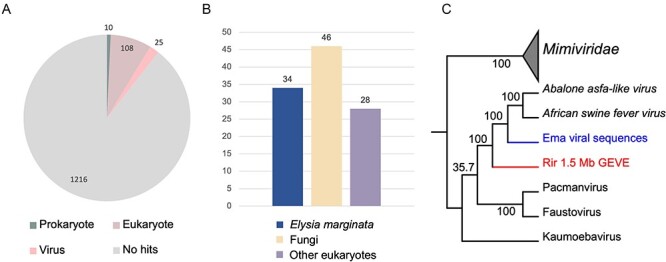
Relationship between the GEVE on chromosome 8 of strain 4401 and viral sequences in *E. marginata*. (A) Taxonomic distribution of the 1359 ORFs in the 1.5 Mb GEVE region according to the best matches revealed by the BLASTp search of the edited NR database. (B) Details regarding 108 eukaryotic annotations. The 34 best matches to *E. marginata* sequences were from one assembly (GCA_019649035.1). (C) Maximum-likelihood phylogenetic tree of the viral sequences from the *E. marginata* genome assembly constructed using three concatenated markers (PolB, RNAPL, and RNAPS). *Mimiviridae* sequences were selected as the outgroup. Rir, *R. irregularis*; Ema, *E. marginata*. The best-fit model was Q.pfam+F+I+G4.

We performed a BLASTp search of the NR database using the 1,359 predicted ORFs in the 1.5-Mb GEVE region as queries. On the basis of the best matches, we determined the most likely taxonomic distribution of 143 ORFs (*E*-value < 10^−5^). More specifically, 108 ORFs were most similar to eukaryotic sequences, 10 ORFs were most similar to prokaryotic sequences, and 25 ORFs were most similar to viral sequences (21 of the viral sequences were from *Asfarviridae*) (Fig. 5A). A similar taxonomic distribution was observed for previously reported nucleocytoviruses lacking close relatives in databases (Blanc-Mathieu et al. 2021).

Notably, 34 of the 108 ORFs that are most closely matched eukaryotic sequences were most similar to a single-genome assembly of a sea slug species (*Elysia marginata*) (Maeda et al. 2021) (Fig. 5B). However, most of the following matches for these ORFs were genes from viruses belonging to *Asfarviridae*, suggestive of the presence of viral-like sequences in the *E. marginata* genome assembly. By screening the *E. marginata* genomic data, we detected thirteen putative viral regions and ten *Nucleocytoviricota* markers (PolB, RNAPS, RNAPL, MCP, RNR, VLTF3, A32, SFII, D5, and mRNAc) (Supplementary Tables S1 and S5). Six regions had a GC content of ∼35 per cent, which was similar to the GC content of *E. marginata* (36.5 per cent), but seven regions had a higher GC content (56.7 per cent). The total length of these seven regions was 124 kb and included all of the identified marker genes. These seven viral regions encompassed the almost entire length of the contigs. The coverage of these contigs is higher (five to sixfold) compared with other contigs (Supplementary Table S5–S7).

A phylogenetic analysis of the marker genes confirmed that the viral sequences in the *E. marginata* genome assembly are related to *Asfarviridae* and the GEVE in *R. irregularis* (Fig. 5C). The viral sequences were positioned between the fungal GEVE and the clade including *African swine fever virus* and *Abalone asfa-like virus*. Consistent phylogenetic relationships were also detected among each marker gene (Supplementary Figs S2–S6, S11). Thus, the *E. marginata* genomic data contains sequences related to *Asfarviridae*.

Besides the thirty-four ORFs similar to sequences in the sea slug assembly, there were seventy-four ORFs in the GEVE region that showed close similarities to eukaryotic homologs in the database. Among them, seventy-three ORFs were homologous to the genes predicted in the other genomic region of strain 4401. We also noted that several regions within the GEVE region showed relatively low viral scores, where eukaryotic-like genes were abundant (Supplementary Fig. S1).

## Discussion

The fungal virosphere, which mostly comprises RNA viruses, lacks obvious dsDNA viruses (Kondo, Botella, and Suzuki 2022). In the present study, we identified a continuous 1.5-Mb *Nucleocytoviricota* GEVE region in the arbuscular mycorrhizal fungus *R. irregularis*. Prior to this study, the longest known continuous GEVE (475 kb) was detected in a genomic contig from a green alga (Moniruzzaman, Erazo-Garcia, and Aylward 2022). Thus, the GEVE region we found in a fungal chromosome represents the longest continuous GEVE in a eukaryotic genome identified to date. Our phylogenetic analysis indicated that this GEVE is closely related to the clade containing *African swine fever virus* and *Abalone asfa-like virus* (Fig. 4B, Supplementary Figs S2–S6). Additionally, the GEVE region includes single copies of five *Nucleocytoviricota* marker genes (Fig. 4A), and their GC content and Hi-C profile differ from those of the other parts of the chromosome (Fig. 1B and D). These results suggest that the GEVE region originated from a single insertion of an *Asfarviridae*-related virus. Compaction of the GEVE genomic region was revealed by the Hi-C data. Compaction of the genome can inhibit the binding of transcription factors or RNA polymerase, thereby suppressing gene expression (Shukron et al. 2019; Boltsis et al. 2021). Therefore, the compaction of the GEVE region may reflect the silencing of genes in this region, possibly representing a defense mechanism of fungi against the invasion of exogenous DNA. The GEVE was detected in only one of the five analyzed strains (Fig. 2, Supplementary Table S3). This GEVE region had a lower repeat and TE density than the other fungal chromosomal regions, implying that the region experienced a shorter time for invasions of TEs than the other chromosomal regions (Fig. 3). This viral integration was probably a relatively recent event, suggesting that there are dsDNA viruses that are still actively infecting the fungal species.

The genomes of isolated *Asfarviridae* viruses (155–466 kb) (Reteno et al. 2015; Matsuyama et al. 2020) are much smaller than the 1.5-Mb GEVE region identified in the fungal chromosome. Furthermore, the coding density of the GEVE region was low (36.22 per cent). Most of the ORFs annotated as eukaryotic origin had homologs in regions elsewhere in the genome of strain 4401. Similarly, there were a few regions enriched in eukaryotic best hits (Supplementary Fig. S1). These indicate a possibility of insertions of eukaryotic genes in some parts of the GEVE regions. The finding of introns in genes of apparently viral origins also suggests the possibility of invasions of introns (Huff, Zilberman, and Roy 2016). The expansion of repeated sequences after the viral integration also accounts for part of the expansion of the GEVE region; repeats represented 18.37 per cent of the non-coding regions. However, these evolutionary events do not appear to fully account for the high proportion of non-coding sequences in the GEVE (63.78 per cent). The substantial part of non-coding sequences in the GEVE is probably originated in former coding sequences (i.e. decaying genes), as one-fourth of the intergenic sequences displayed traces of genes (Supplementary Fig. S9). A part of these decaying genes appears to be of viral origins, but it was difficult to determine the origin (either viral or fungal) for the others. Overall, the secondary insertion of fungal sequences after viral integration could be an important factor to the giant size of GEVE region.

Although the long and contiguous GEVE region was detected only in chromosome 8 of strain 4401, *polB* genes that may have originated in *Asfarviridae* were identified on multiple chromosomes in all strains (Supplementary Table S1). The genomic positions and copy numbers of these *polB* genes varied among the genomes of the analyzed *R. irregularis* strains (Supplementary Table S4). This suggests that infections of *R. irregularis* by *Asfarviridae*-like viruses may be a widespread and ongoing event.

*Asfarviridae* viruses infect a variety of eukaryotes, such as swine, abalone, and amoebae, although only five have been isolated and completely sequenced (Karki, Moniruzzaman, and Aylward 2021). Previous studies have identified marine dinoflagellates (Ogata et al. 2009) and oomycetes (Hannat et al. 2021) as potential hosts of *Asfarviridae*. In the present study, we detected *Asfarviridae*-like genome sequences closely related to the fungal GEVE within the *E. marginata* genome assembly (Fig. 4C). The viral sequences in the *E. marginata* genome assembly encode all of the core genes (Supplementary Table S1), suggesting that the virus containing these sequences can form virion and is infectious. Unlike the GEVE in *R. irregularis*, the seven viral regions with high GC contents in *E. marginata* covered almost the entire length of their contigs and have obviously distinct sequence coverage, suggesting that these viral regions are not insertions in the sea slug genome but were derived from viral genomes concomitantly sequenced with the *E. marginata* assembly (Supplementary Table S5–S7). Although our current data are insufficient to conclude that they are viruses infecting the sea slug, these findings indicate that *Asfarviridae* viruses are likely more widespread and diverse than currently recognized.

Recent research confirmed that MCP is one of the major components of virions (Krupovic, Makarova, and Koonin 2022), but no MCP gene was detected in the *R. irregularis* genome (Supplementary Table S1). However, we identified *Asfarviridae* minor capsid protein and H240R encoding genes in the 1.5-Mb GEVE region, implying that this virus may have produced virions. There are two possible explanations for the lack of MCP genes. First, some genomic regions may have been deleted or genomic rearrangements might have occurred after the viral genome was integrated (Moniruzzaman et al. 2020), resulting in a lack of an MCP gene in the GEVE region. Second, the virus from which the GEVE was derived may use a major virion protein whose sequence substantially differs from known MCPs so that our method based on reference sequences failed to identify it. The MCPs of nucleocytoviruses are highly diverse, and some nucleocytoviruses (e.g. pandoraviruses) are known to use different types of proteins as the major components of the virion (Krupovic, Yutin, and Koonin 2020).

Arbuscular mycorrhizal fungi are obligate plant-mutualistic organisms that provide significant benefits to plants (e.g. increased nutrient levels and enhanced disease resistance) (Gosling et al. 2006). As a model organism of arbuscular mycorrhizal fungi, *R. irregularis* forms a robust tripartite association with its endobacteria and plants during its life cycle; horizontal gene transfers among these organisms contribute to their evolution and symbiotic adaptation (Li et al. 2018). For example, foreign genes affect *R. irregularis* life cycle–related processes, including gene expression, mitosis, and signal transduction (Li et al. 2018). In the present study, we revealed that dsDNA viruses may also be important for the horizontal transfer of genes in *R. irregularis*. Future functional analyses of these virus-derived genes in *R. irregularis* may provide novel insights into the ecology and evolution of this beneficial microorganism.

## Methods

### Detection of viral regions

To identify virus-like regions in eukaryotic genomes, we used ViralRecall v2.1 (Aylward and Moniruzzaman 2021) to screen genomic data, with a window size and viral score set at 150 ORFs and 0, respectively (-s 0 -w 150). ViralRecall is a tool designed for identifying virus-like regions. Notably, this tool uses *Nucleocytoviricota* orthologous groups and the Pfam database to detect *Nucleocytoviricota* signals. This tool evaluates the viral and cellular scores for each ORF in the genome and determines the likelihood that these regions are virus-like. Chromosome-level genomic data for five *R. irregularis* strains were retrieved from GenBank, whereas *E. marginata* genomic data were retrieved from GCA_019649035.1 (Maeda et al. 2021). The GC content was calculated using in-house Python scripts, with the window size set at 50,000 bp to minimize the effect of repeats. The insertion of the viral region in the fungal chromosome was validated as follows. First, long reads of strain 4401 (accession no. SRR15461860) were mapped to the whole genome using Minimap2 v2.24 (Li and Birol 2018). We applied the ‘view’ function of Samtools v1.16.1 (Li et al. 2009) to select reads connecting the virus and host region. The ‘bamtobed’ function of Bedtools v2.29.2 (Quinlan and Hall 2010) was used to visualize the results. The comparison of chromosome 8 from different strains (at the amino acid sequence level) was performed using DiGAlign v1.3 (http://www.genome.jp/digalign/). The coverage of contigs in the *E. marginata* assembly was calculated by the same method as above, using DRR238952 as the raw data.

### Generation of Hi-C contact map and detection of repeats

To clarify the structure of the chromosome with the 1.5-Mb GEVE region, we transformed the raw Hi-C sequencing data of strain 4401 from the NCBI Sequence Read Archive (accession no. SRR15461854) into normalized contact maps using Juicer v2.0 (Durand et al. 2016) and visualized the results using Juicebox (http://aidenlab.org/juicebox/). Repetitive elements in the genome were identified and masked using RepeatModeler v2.0.2 (Flynn et al. 2020) and RepeatMasker v4.1.2 (http://www.repeatmasker.org). RepeatModeler can identify repeats (in both coding and non-coding regions) and annotate TEs, including retroelements and DNA transposons, with distinct discovery algorithms. We used the ‘-LTRStruct’ parameter while running RepeatModeler to detect long terminal repeat retroelements.

### Phylogenetic analyses

*Nucleocytoviricota* marker genes were predicted using the built-in HMMER profile of ViralRecall. To verify whether these genes are indeed viral homologs, we obtained reference sequences of these marker genes from a previous study (Kazlauskas et al. 2020) and GenBank and then constructed phylogenetic trees as follows. A multiple sequence alignment was completed using Clustal-Omega v1.2.4 (Sievers and Higgins 2018) and trimmed using trimAl v1.4.1 (parameter: ‘-gt 0.1’) (Capella-Gutiérrez, Silla-Martínez, and Gabaldón 2009). Maximum-likelihood phylogenetic trees were generated using IQ-TREE v2.2.0 (Minh et al. 2020), with 1,000 ultrafast bootstrap replicates (Hoang et al. 2018). The best-fit model was selected using ModelFinder (Kalyaanamoorthy et al. 2017). Phylogenetic trees were visualized using iTOL v6.7.4 (Letunic and Bork 2019).

We screened the genome of strain 4401 for MCP genes using hmm files constructed from the MCP sequences of phages, *Nucleocytoviricota, Mirusviricota*, and *Herpesvirales,* as well as HMMER v3.3.2 (Eddy and Pearson 2011) (*e* < 0.05). We also searched for major virion capsid 1 and 2 of pandoraviruses using the same method. Reference sequences of phages, *Herpesvirales*, pandoraviruses, and other *Nucleocytoviricota* viruses were obtained from GenBank and the NCBI protein database. Previously reported MCP sequences of *Mirusviricota* were also used (Gaïa et al. 2023). Considering the possibility of missing results, if a sequence in the fungal genome is not identified as an ORF, we used BLAST+ to perform tBLASTn searches (Camacho et al. 2009) of the nucleotide database of these five fungal genomes to identify MCP sequences (*E*-value < 0.05).

### Annotation of viral regions

To predict ORFs in the 1.5 Mb GEVE, we first used Prodigal v2.6.3 (Hyatt et al. 2010) with the default parameter. Considering the potential pseudogenization happened in the viral region, we also translated all regions between STOP codons in the same frame (STOP-to-STOP ORFs) by using EMBOSS:getorf (https://www.bioinformatics.nl/cgi-bin/emboss/getorf). The STOP-to-STOP ORFs contain almost all information on the potential coding sequences that exist or used to exist on the GEVE region, and we removed the predicted result which largely (>50 per cent) overlapped with the prodigal result. We annotated these ORFs (>50 amino acids) by using Diamond v2.0.15 (Buchfink, Xie, and Huson 2015) search against the RefSeq database (--ultra-sensitive, *E*-value < 10). To identify pseudogenes and intron-containing genes, we investigated the sets of neighboring ORFs with the same orientation and with the same database hits. Then, we compared the nucleotide sequences covering these ORFs to the amino acid sequences of the reference sequences in RefSeq by using Dotter v4.22 (Sonnhammer and Durbin 1995) to make dot-plots. On the one hand, some of such neighboring ORFs were identified as candidates of genes with introns, when the ORFs are continuously aligned with the reference sequences at the protein sequence level. These regions were further examined with FGENESH v2.6 (Solovyev et al. 2006) to predict exons. When the program predicted two or more exons, the regions were assumed to contain genes with introns. On the other hand, many other cases were identified as pseudogenes. To maximize the gene annotation, we also incorporated the original fungal gene annotations (Yildirir et al. 2022) for some GEVE regions, that is, the regions with either (1) genes with introns as identified above or (2) with no predicted genes or pseudogenes by the above procedure. In the case of (1), we overwrite the above gene annotations with the original fungal annotations. In the case of (2), we added the original fungal annotations.

The ORFs were annotated using BLASTp in Diamond (*E*-value < 10^−5^). Because previous studies may have annotated viral insertions in fungal genomes as fungal genes, we used the NR database excluding the sequences from the fungal class Glomeromycetes (NCBI: txid214506, which includes *R. irregularis*). In annotating the sea slug viral regions, we used the NR database and excluded all sequences of *E. marginata* (NCBI: txid1093978). The best match for each ORF was used to determine the taxonomic distribution of the ORFs (i.e. eukaryote, prokaryote, and virus). For ORFs with the eukaryotic best hit (excluding *E. marginata*), we performed BLASTp to search against other predicted ORF in strain 4401 except for the 1.5-Mb viral region. Functional annotations were retrieved using eggNOG-mapper v2.1.9 (Cantalapiedra et al. 2021).

To identify traces of genes, we extracted the genomic region between two predicted ORFs and eliminated the regions shorter than 100 bp before performing a BLASTx search using Diamond (*E*-value < 10^−5^). The NR database and all predicted proteins on viral regions identified by ViralRecall were used as the reference and ‘--ultra-sensitive’ was selected as the parameter. We also used Tandem Repeat Finder v4.09 (Benson 1999) to identify the tandem repeats in the GEVE region, with ‘2 7 7 80 10 30 2000 -f -d -m’ selected as the parameter.

## Supplementary Material

vead064_Supp

## Data Availability

The sequence of the 1.5-Mb GEVE region and all multiple sequence alignments used in phylogenetic trees are available in the Supplementary Dataset as well as via GenomeNet (https://www.genome.jp/ftp/db/community/Fungal_GEVE).
