# Supplementary material for: A 1.5-Mb continuous endogenous viral region in the arbuscular mycorrhizal fungus Rhizophagus irregularis
Source: Virus Evol. 2023 Oct 31;9(2):vead064. doi: 10.1093/ve/vead064 (PMC10640383; doi:10.1093/ve/vead064)
Supplement: vead064_Supp [file vead064_supp.zip › SupplementaryFigs.pdf]

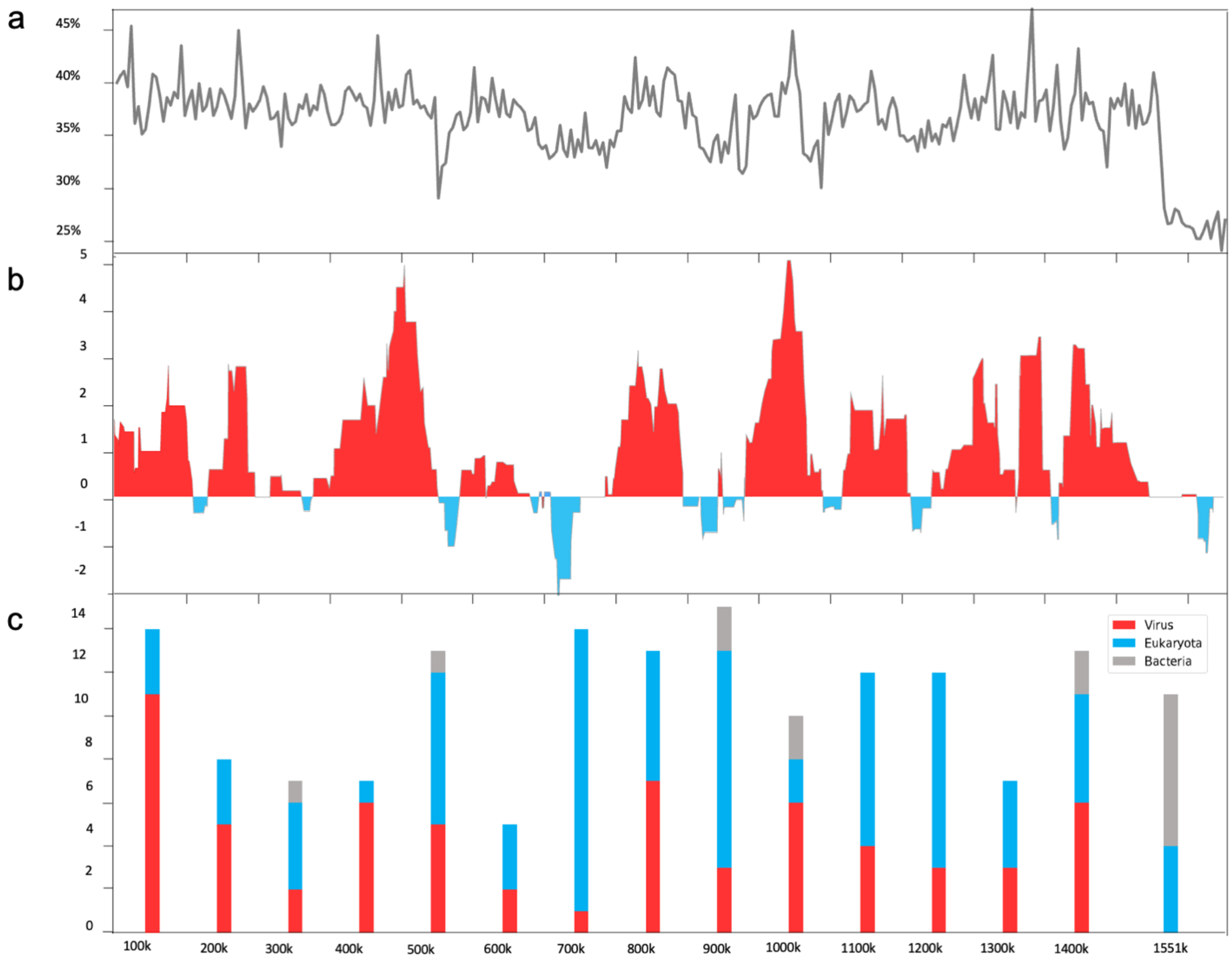

Supplementary Fig S1: Details regarding the 1.5 Mb GEVE region with small window sizes. (a) GC content along the viral region. The window size is 5,000 bp. **(b) ViralRecall score of different parts in this viral region.** Viral scores were evaluated with a rolling window of 15 ORFs on the viral region. **(c) Taxonomic distribution of annotated sequences in each 100 kb on this viral region.** The annotated sequences here including both genes and pseudogenes.

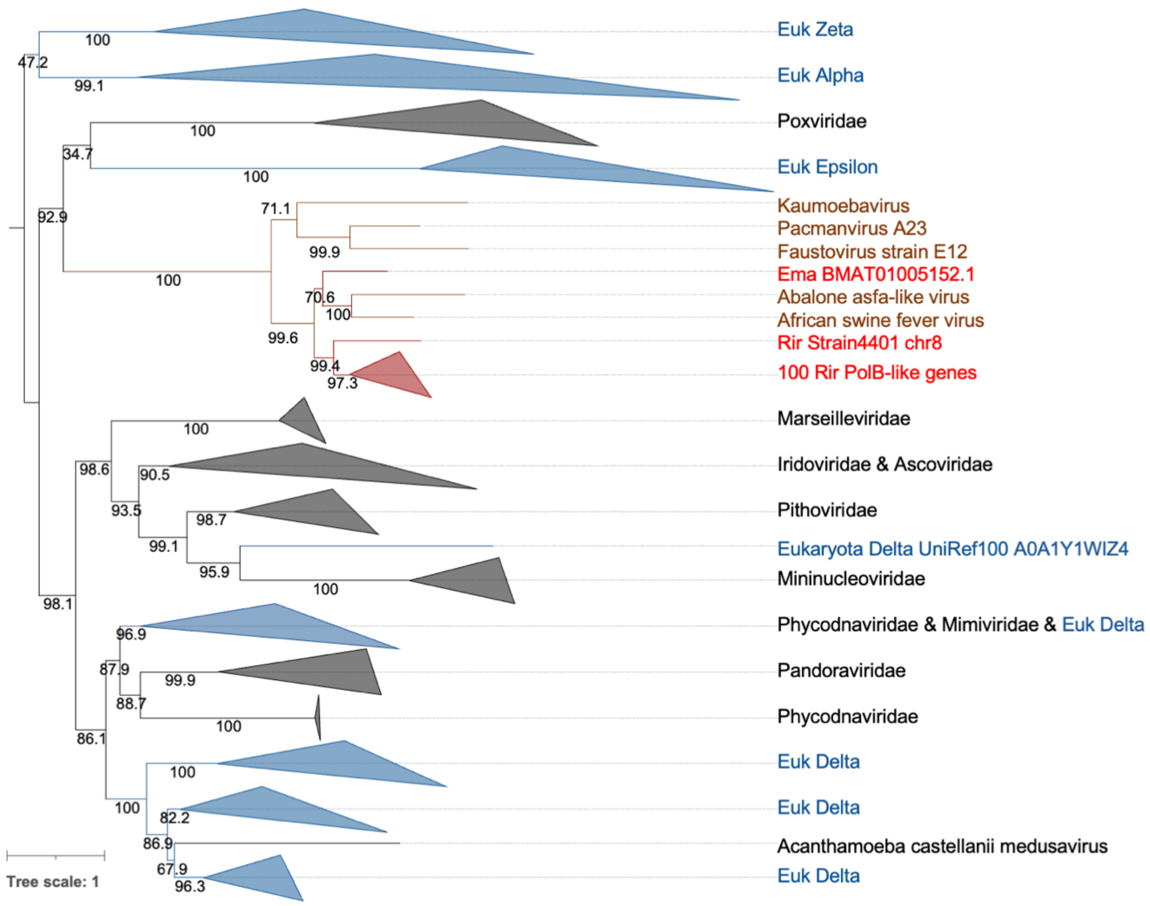

Supplementary Fig S2: Phylogenetic tree of 101 PolB candidates predicted by ViralRecall. Clades containing eukaryotic sequences are in blue. *Asfarviridae* sequences are in brown. Viral sequences from *R. irregularis* and *E. marginata* are indicated by red labels. Ultrafast bootstrap support values are provided along the branches. These 100 PolB sequences are phylogenetically distinct from the one detected in the 1.5 Mb GEVE region. The root of the tree was arbitrarily chosen and the tree should be considered as an unrooted tree. The best-fit model was Q.pfam+F+R10.

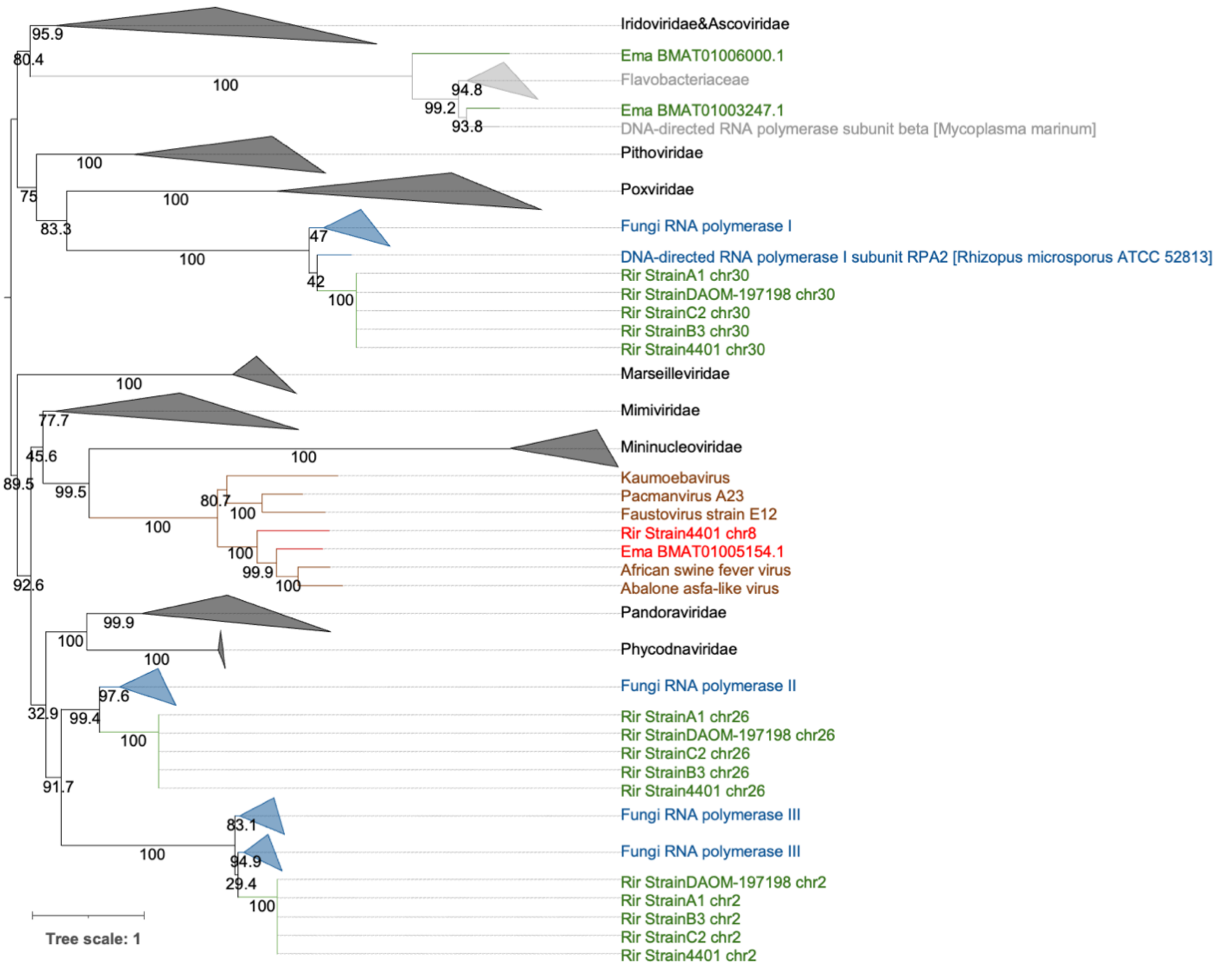

Supplementary Fig S3: Phylogenetic tree of RNAPS candidates predicted by ViralRecall. The Fig. S2 legend explains the meaning of the different colors; however, eukaryotic and bacterial sequences in *R. irregularis* and *E. marginata* are in green and bacterial sequences are in light gray. Ultrafast bootstrap support values are provided along the branches. The root of the tree was arbitrarily chosen and the tree should be considered as an unrooted tree. The best-fit model was LG+F+I+I+R8.

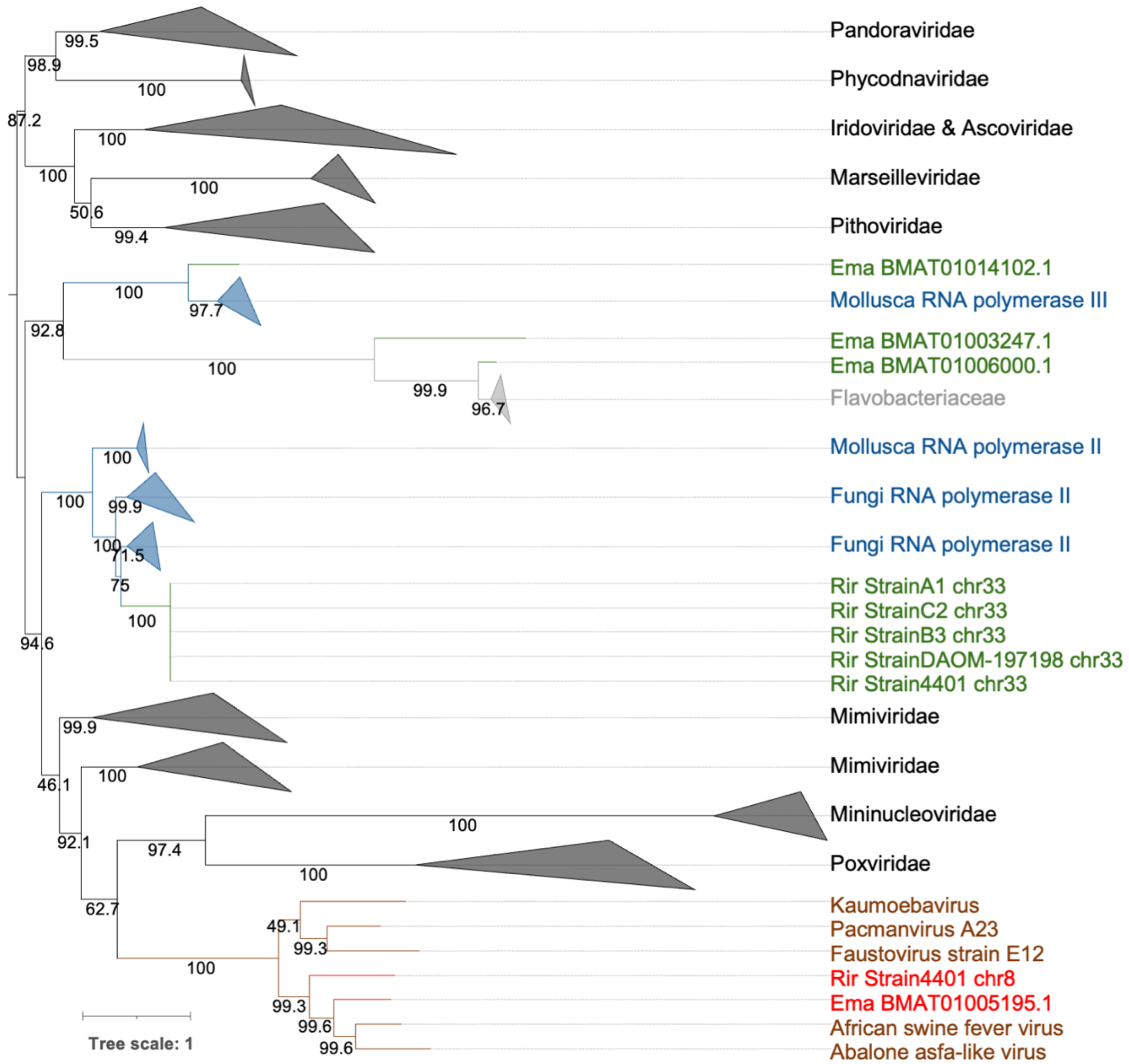

Supplementary Fig S4: Phylogenetic tree of RNAPL candidates predicted by ViralRecall. The Fig. S3 legend explains the meaning of the different colors. Ultrafast bootstrap support values are provided along the branches. The root of the tree was arbitrarily chosen and the tree should be considered as an unrooted tree. The best-fit model was LG+F+R10.

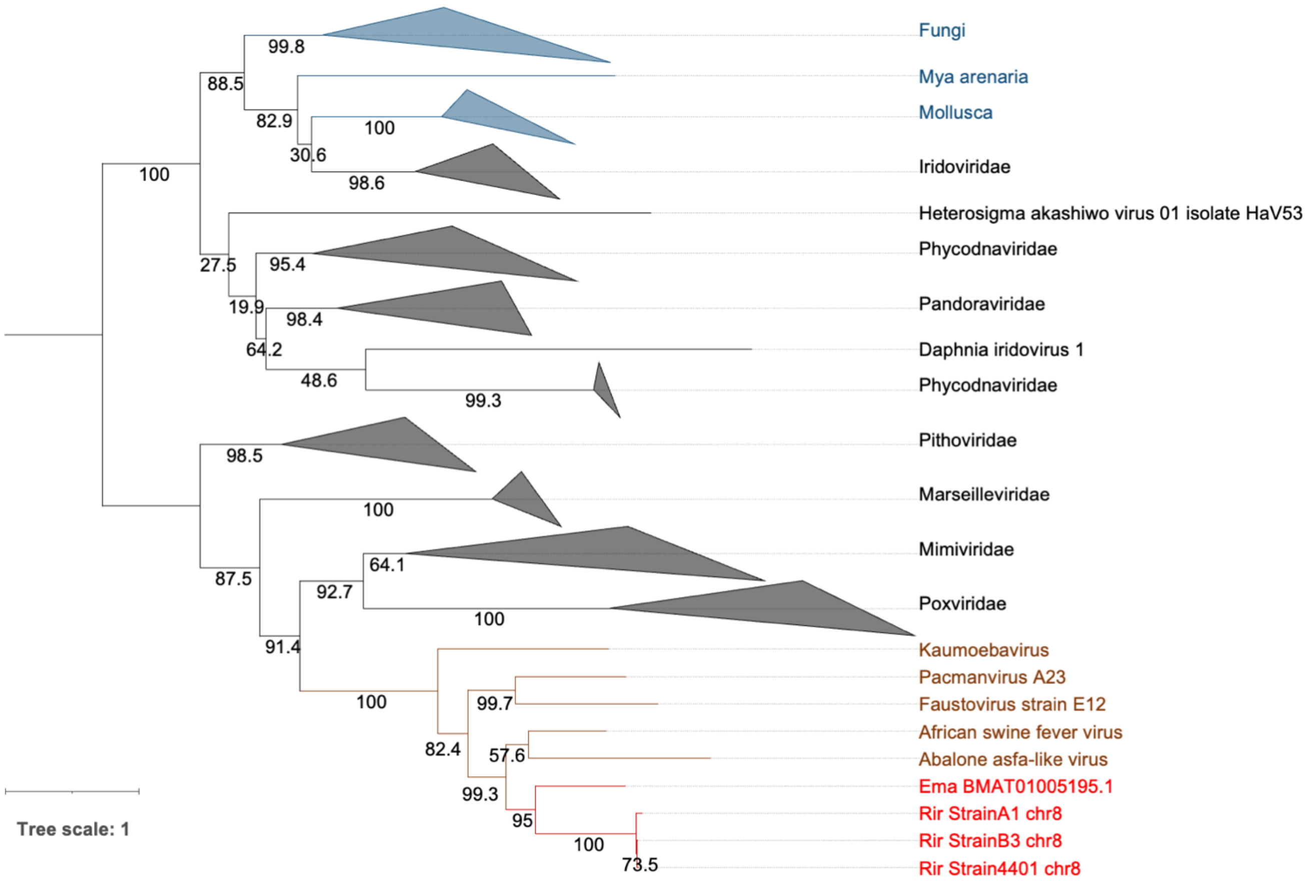

Supplementary Fig S5: Phylogenetic tree of mRNAc candidates predicted by ViralRecall. The Fig. S3 legend explains the meaning of the different colors. Ultrafast bootstrap support values are provided along the branches. The root of the tree was arbitrarily chosen and the tree should be considered as an unrooted tree. The best-fit model was Q.pfam+F+I+I+R6.

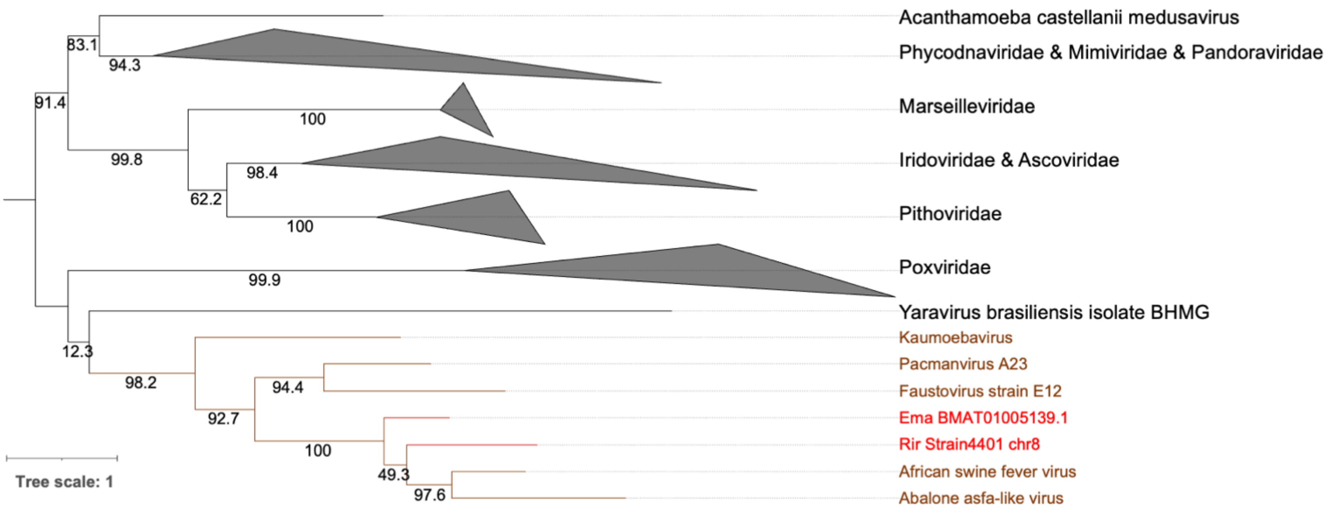

Supplementary Fig S6: Phylogenetic tree of VLTF3 candidates predicted by ViralRecall. The Fig. S2 legend explains the meaning of the different colors. Ultrafast bootstrap support values are provided along the branches. The root of the tree was arbitrarily chosen and the tree should be considered as an unrooted tree. The best-fit model was Q.insect+F+R6.

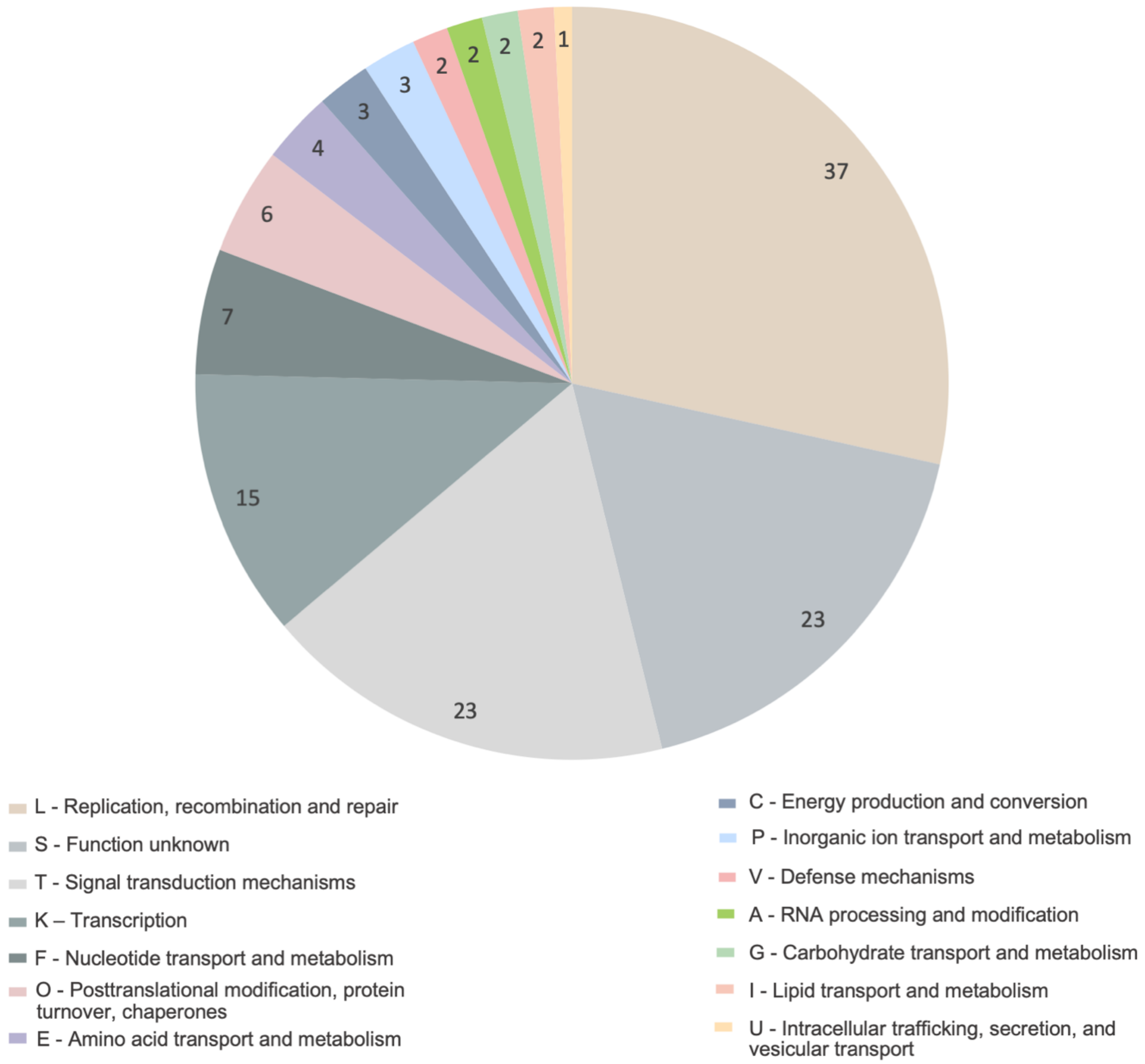

Supplementary Fig S7: Functional annotation of the GEVE. The number of ORFs belonging to each COG functional category is indicated.

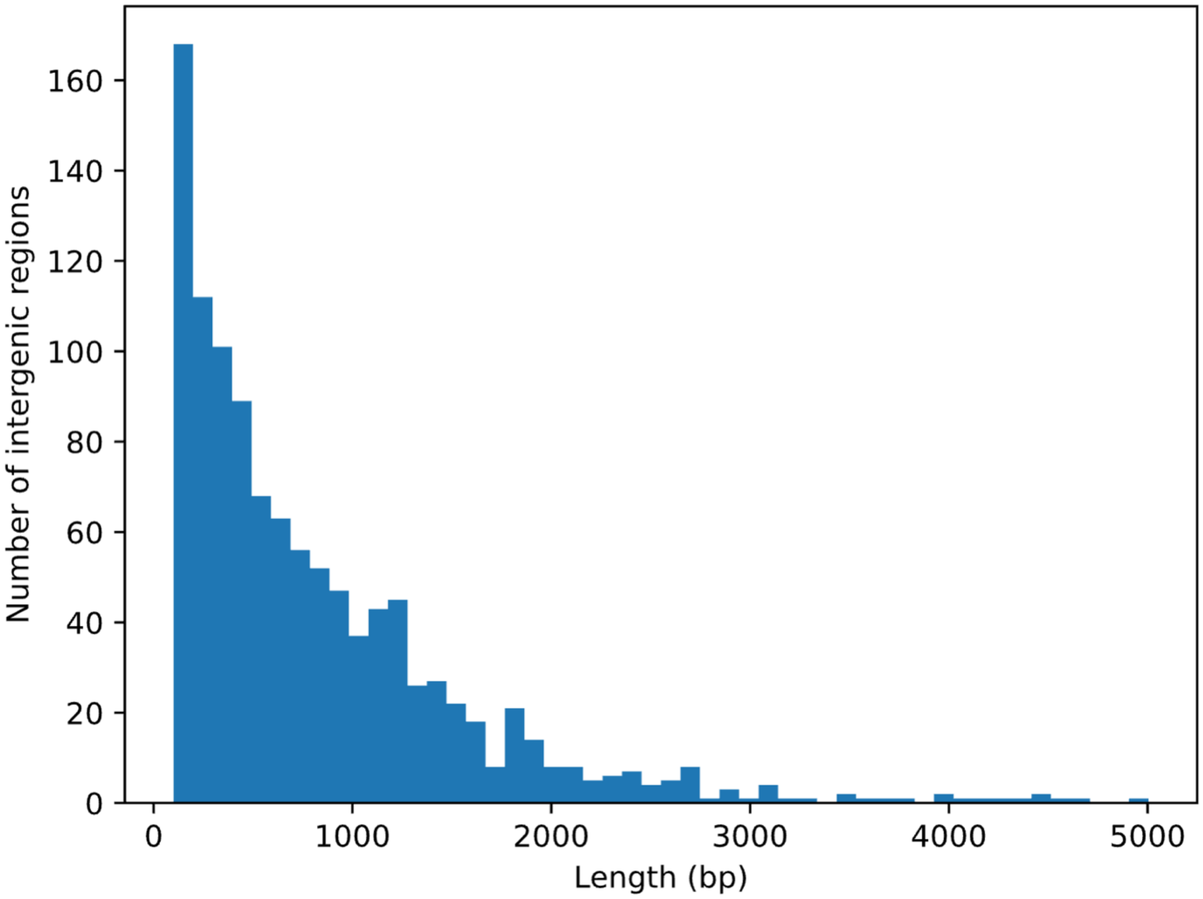

Supplementary Fig S8: Lengths of the 1095 intergenic sequences in the 1.5 Mb GEVE region. The longest intergenic sequence is 5,004 bp. The average length and standard deviation are 828 bp and 743 bp, respectively.

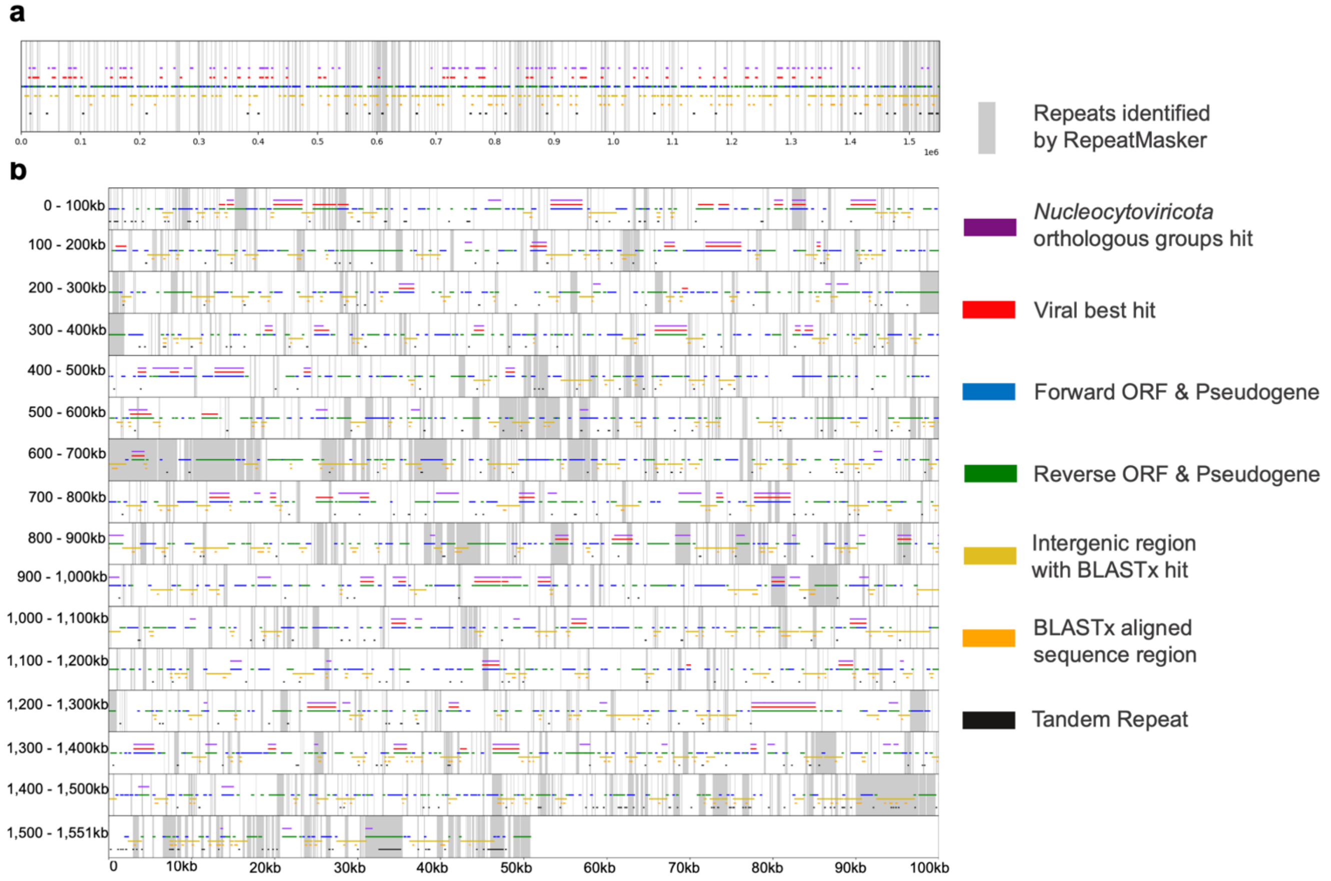

Supplementary Fig S9: Distribution of genomic components in the 1.5 Mb GEVE region. (a) Overall distribution of genomic components in the GEVE region. Gray vertical lines represent repeats identified by RepeatMasker. Blue and green horizontal lines represent the forward and reverse annotated sequences (i.e., genes or pseudogenes), respectively. The red horizontal line represents a sequence that is most similar to a viral sequence (sequences from *E. marginata* were also considered as viral sequences), whereas the purple horizontal line represents a match with *Nucleocytoviricota* orthologous groups in ViralRecall. The yellow horizontal line represents the intergenic region with matched sequences according to the BLASTx results (i.e., traces of genes), whereas the orange horizontal line represents the precise locations of BLASTx aligned sequence regions. The black horizontal line represents the tandem repeat region identified by Tandem Repeat Finder. **(b) Details regarding the distribution of genomic components in the 1.5 Mb GEVE region.** The colors are the same as in (a). Each row represents 100 kilobases. Some ORFs were identified as repeats by RepeatMasker because of the existence of similar sequences in the fungal genome.

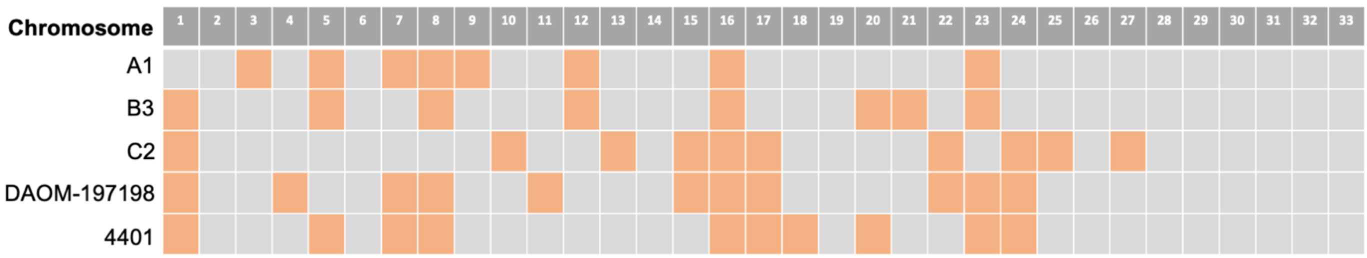

Supplementary Fig S10: Distribution of viral regions on chromosomes of the five fungal strains. The orange background grid indicates that the viral region has been detected on this chromosome, while the gray background represents that no viral region has been detected.

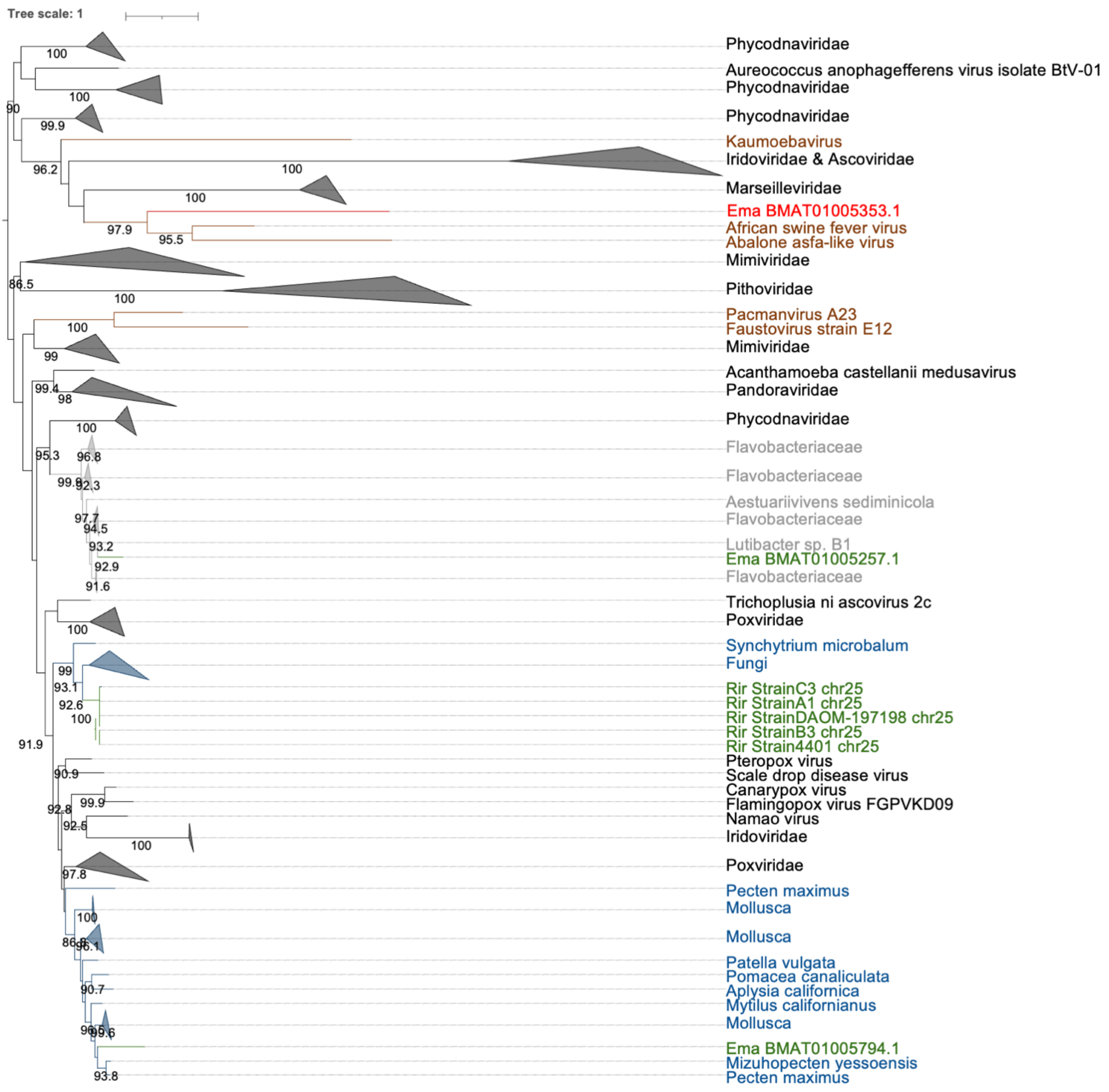

Supplementary Fig S11: Phylogenetic tree of RNR candidates predicted by ViralRecall. The Fig. S3 legend explains the meaning of the different colors. Ultrafast bootstrap support values are provided along the branches. The root of the tree was arbitrarily chosen and the tree should be considered as an unrooted tree. The best-fit model was Q.yeast+I+I+R7.
